# Traditional Chinese medicine combined with Moxibustion in the treatment of “long-COVID”: A protocol for systematic review and meta-analysis

**DOI:** 10.1097/MD.0000000000031447

**Published:** 2022-10-28

**Authors:** Dongqiang Luo, Bin Liu, Pengxin Wang, Hui Liao, Shuxian Mao, Huicong Chen, Yuxin Huang, Lu Liu, Wanning Lan, Feng Liu

**Affiliations:** a Guangzhou University of Chinese Medicine, Guangzhou, China; b Clifford Hospital, Guangzhou, China.

**Keywords:** meta-analysis, moxibustion treatment, protocol, systematic review, traditional Chinese medicine treatment

## Abstract

From the end of 2019 to now, COVID-19 is still prevalent, which poses a great threat to international public health. With the increasing number of people infected, the number of patients with COVID-19 sequelae is also increasing, but there is no specific drug for COVID-19 sequelae. In China, traditional Chinese medicine combined with acupuncture has been widely used in COVID-19 sequelae, but there is still a lack of evidence-based medicine evaluation. The purpose of this study was to evaluate the efficacy and safety of traditional Chinese medicine combined with moxibustion in the treatment of COVID-19 sequelae.

**Methods:**

According to the retrieval strategy, the “long COVID” randomized controlled trial of traditional Chinese medicine combined with moxibustion will be search in eight databases composed of PubMed, Embase, Web of Science, China National knowledge Infrastructure Database, China Biomedical Database and China Science and Technology Journal Database, regardless of publication date or language. The study was screened according to the inclusion and exclusion criteria, and the Cochrane risk bias assessment tool was used to evaluate the quality of the study. Meta-analysis was carried out using RevMan5.3 and STATA12.0 software. Finally, the level of evidence of the results will be evaluated.

**Results:**

This study will evaluate whether traditional Chinese medicine combined with moxibustion can effectively treat the symptoms of COVID-19 sequelae.

**Conclusion:**

This study will provide evidence whether there is benefit of traditional Chinese medicine combined with moxibustion in the treatment of COVID-19 sequelae. At the same time, our research results will provide a reference for clinical decision-making and guiding development in the future.

## 1. Introduction

COVID-19 refers to a disease after infection with novel coronavirus.^[1]^ It has been more than two years since the first case of COVID-19 was reported in Wuhan, China in late December 2019. However, at present, the global epidemic situation is still grim. Recent evidence suggests that a series of symptoms persist after the acute infection. This is a condition known as long COVID.^[2]^ while the number of people infected with novel coronavirus is increasing, the number of patients with “long COVID-19” is also increasing, which poses a great threat to international public health.^[3]^

Clinical observation shows that most clinical cured patients still have different symptoms after recovery. As acute COVID-19, long COVID can involve multiple organs and affect many systems, including, but not limited to, respiratory, cardiovascular, neurological, gastrointestinal and musculoskeletal systems. COVID-19’s long-term symptoms include fatigue, dyspnea, heart abnormalities, cognitive disorders, sleep disorders, post-traumatic stress disorder symptoms, muscle pain, attention problems and headaches. Statistics shows that among these symptoms, the symptoms of nervous system and cardiovascular system are the most, and seriously affect the quality of life of patients.^[4]^ At present, there is no specific drug to treat “long COVID-19.”^[4,5]^ The treatment of “long COVID-19” in modern medicine mainly use of drugs to improve related symptoms, combined with multi-disciplinary rehabilitation model and telerehabilitation. Research shows that traditional Chinese medicine combined with moxibustion has a good effect on improving the symptoms of patients with chronic coronavirus disease.^[6,7]^ But there is still a lack of articles to systematically explore the treatment of “long COVID-19” with traditional Chinese medicine combined with acupuncture.^[8]^ Therefore, we are going to carry out this systematic review and meta-analysis in order to provide new ideas for clinical diagnosis and treatment.

## 2. Methods and analysis

### 2.1. Objectives and registration

The systematic review and meta-analysis will be conducted to summarize the evidence and evaluate the effect of traditional Chinese medicine combined with moxibustion on the improvement of symptoms in patients with long-COVID. This protocol has been registered with PROSPERO (No.CRD42022351277)(https://www.crd.york.ac.uk/PROSPERO/). The PRISMA-P 2015 statement and the updated guideline in 2020 will be followed for reporting the study.^[9,10]^

### 2.2. Eligibility criteria

#### 2.2.1 Type of studies.

Merely randomized controlled trials are included without the limit of language and publication date. It will be excluded that if the article belongs to observational study, animal experimental research, review, study of different methods, duplicate references or whose data is missing.

#### 2.2.2 Type of participants.

Patients with long-COVID include: Individuals with symptoms of COVID-19 within 4 to 12 weeks after the onset of acute symptoms; Individuals with post symptoms of COVID-19 within 4 to 12 weeks after the onset of acute symptoms over 12 weeks after the onset of acute symptoms. It is excluded if participants have symptoms similar to long-COVID but test negative for novel coronavirus. The common symptoms in long-COVID consist of cough, fatigue, headache, loss of smell, loss of memory, taste disorders but more varied.

#### 2.2.3 Type of interventions.

The measures in experimental group should be traditional Chinese medicine combined with moxibustion based on the conventional therapy. Due to the principle of individual-oriented, there is no restriction on the technique, acupoints, time, frequency and cycle of moxibustion as well as the type, dose, course, medication time and frequency of traditional Chinese medicine. Meantime, the control treatment ought to be the conventional therapy.

#### 2.2.4 Type of outcome measurements.

The primary outcome emphasizes total clinical effective rate. Secondary outcomes comprise the speed of relieving, TCM symptom score, quality of life score, exercise capacity, cognitive ability score and lung function and adverse reactions.

### 2.3. Information sources and search strategy

First, eight major databases which consists of PubMed, Embase, Web of Science, the Cochrane Central Register of Controlled Trials, Chinese National Knowledge Infrastructure database, Chinese Biomedical Database, Chinese Science and Technology Periodical database, and the WanFang database will be searched to find relevant literature using a combination of the main search terms “Long Covid,” “traditional Chinese medicine,” and “moxibustion” within the restriction limit of “randomized controlled trial.” The complete search strategy in Pubmed is displayed in Table 1 as supplementary material. Second, abstracts of ongoing RCTs on patients with long COVID from several of the most important clinical trial registration platform and international conferences will be inspected with the aim of keeping track of the latest research. Last, the reference lists of valuable articles will be examined for additional relevant articles.

**Table 1:** Search strategy for the PubMed database.

Number	Search items
#1	(“post-acute COVID-19 syndrome”[Title/Abstract]) OR (“long-COVID”[Title/Abstract])) OR (“long-haul COVID”[Title/Abstract])) OR (“post-acute COVID syndrome”[Title/Abstract])) OR (“persistent COVID-19”[Title/Abstract])) OR (“post-acute COVID19 syndrome”[Title/Abstract])) OR (“long hauler COVID”[Title/Abstract])) OR (“long COVID”[Title/Abstract])) OR (“post-acute sequelae of SARS-CoV-2 infection”[Title/Abstract])) OR (“long haul COVID”[Title/Abstract])) OR (“chronic COVID syndrome”[Title/Abstract])
#2	(“Traditional Chinese Medicine”[Title/Abstract]) OR (“Chinese Traditional Medicine”[Title/Abstract])) OR (“Chinese Herbal Drugs”[Title/Abstract])) OR (“Drugs, Chinese Herbal”[Title/Abstract])
#3	(moxibustion[Title/Abstract]) OR (moxabustion[Title/Abstract])
#4	Randomized Controlled Trial[Filter]
#5	#1 and #2 and #3 and #4

### 2.4. Study selection

**Figure 1.**
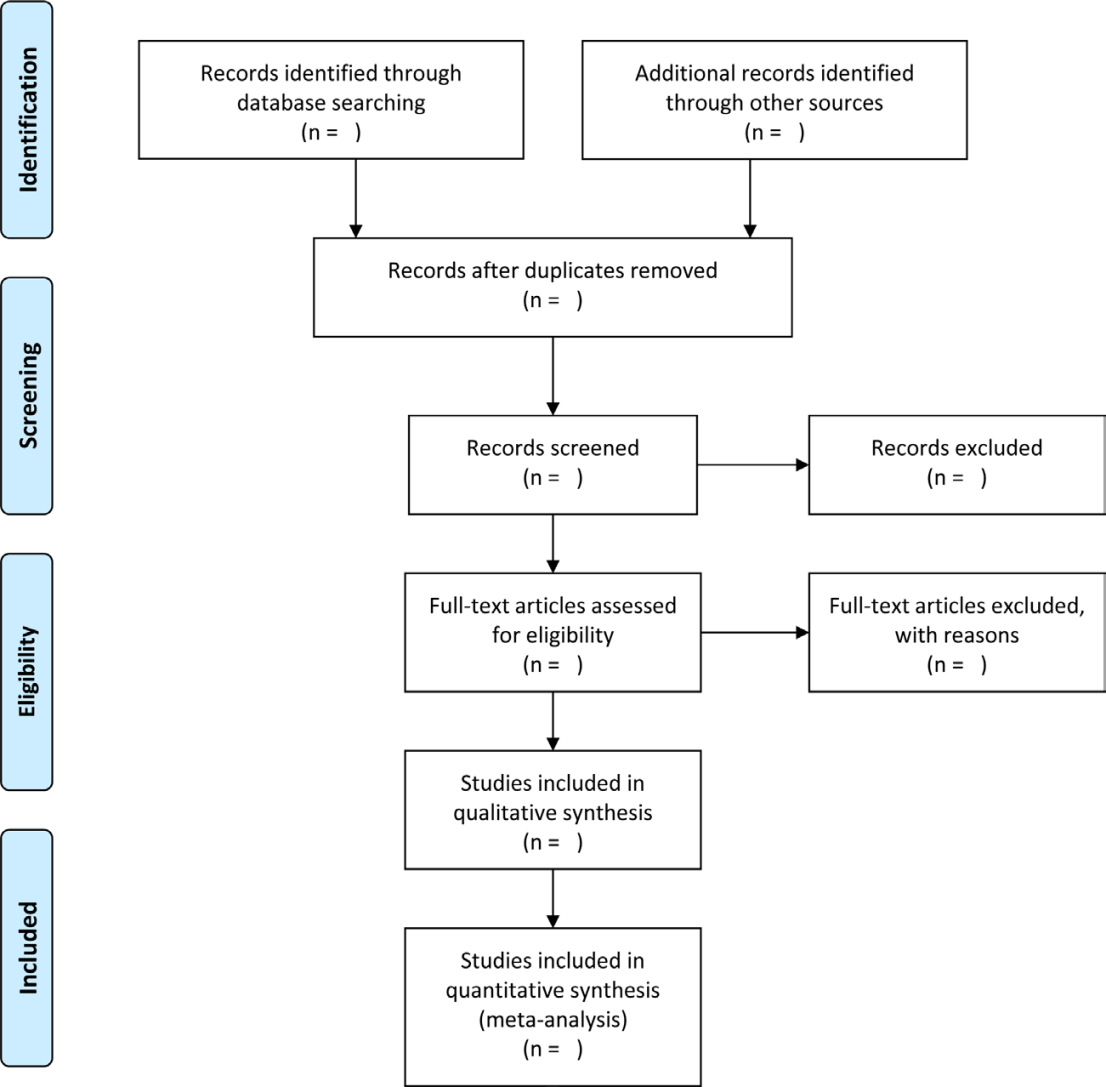
PRISMA flow chart of study selection process.

Study selection will be carried out by two independent experienced researchers. Endnote X9 acts as a literature management tool for retrieved articles. After the software automatically removes duplicate references, they will screen preliminarily the title and abstract to determine whether inclusion criteria are met. Subsequently the selected literature will be downloaded in full text for more detailed screening. In case of controversy, a third investigator will join the discussion. All of the excluded articles will be marked with reasons. The whole selection process will be presented in a PRISMA flow diagram (Fig. 1).^[11]^

### 2.5. Data extraction

Two researchers will separately extract the data from the included studies, including title, first author, published time, sample size, course of disease, symptoms, age, gender, intervention, outcome, adverse events and other required data. Any differentiation will be discussed and arbitrated by the other experienced reviewer. Data on trial details will be collected via Excel2018. *Clinical Trials.gov* and other available sources will be researched for complete data.

### 2.6. Risk of bias in assessment

Risk of bias will be assessed according to the Cochrane Risk of Bias Tool which bases on the following domains: random sequence generation, allocation concealment, blinding of participants and personnel, blinding of outcome assessment, incomplete outcome data, selective outcome reporting, and other sources of bias. Items were scored as low, high, or unclear risk of bias. Two independent researchers will attend the evaluation which will be cross-checked by a third senior one.

### 2.7. Strategy for data synthesis

RevMan 5.3 will be used to perform meta-analysis. Heterogeneity will be tested according to different aspects. The indexes include *Q* test and *I^2^*. *P* > .05 and *I^2^* < 50% indicates small statistical heterogeneity, and fixed effect model will be adopted. Instead, adopt the random effect model. When the outcome variables were dichotomous data, odds ratio and 95% confidence interval will be used. As well mean difference and 95% confidence interval will evaluate the continuous data. For sensitivity analysis, we will turn to Stata version 12.0.

### 2.8. Assessment of heterogeneity

If high heterogeneity is assessed relying on the *P* value and *I^2^* index, subgroup analysis and sensitivity analysis will be performed. Subgroup analysis can be sectionalized by age, the main syndrome, course, interventions, control measures and the study quality. As well to measure the reliability and robustness of the results, we will employ the sensitivity analysis by means of leave one out in Stata. Qualitative synthesis will be adopted if quantitative synthesis is not suitable.

### 2.9. Reporting biases assessment

Since the risk of bias may be affected by publication bias and selective reporting, we will draw the inverted funnel plot in the condition of the number of included literature in no less than ten.

### 2.10. Ethics and dissemination

There is no demand for ethical approval on account of extracted data from published literature, which does not involve patient privacy.

## 3. Discussion

Long covid, which can affect respiratory, cardiovascular and musculoskeletal systems, is a complex multifactorial disease.^[12]^ Although thousands of patients developed “mild” Covid-19 symptoms that did not require hospitalization, a large proportion of them had sequelae after Covid-19.^[13]^ Existing evidence shows that one in five people diagnosed with novel coronavirus infection, regardless of the severity of their acute infection, may have symptoms lasting for 5 weeks or longer, while one in ten people may have symptoms lasting for 12 weeks or longer.^[14]^

Moxibustion therapy is to ignite the moxa stick or moxa stick, stimulate the acupoints of the human body with warm effect through its thermal penetration, and achieve the effect of disease treatment and prevention through the overall action of the meridians and acupoints. Although moxibustion and traditional Chinese medicine are often used for covid-19 in clinical practice, there is no systematic study to prove the effectiveness of moxibustion combined with traditional Chinese medicine in treating covid-19.

Ultimately, we still hope that this study can provide the best possible drug selection and reliable evidence-based medicine for clinical practice, and prove the significant advantages of traditional Chinese medicine in treating “long covid” to a certain extent, and provide more reliable reference value for clinical practice. In addition, we suggest that more large-scale, high-quality and strict randomized controlled trials should be carried out.

## Author contributions

Feng Liu is the guarantor of the article and will be the arbitrator when meeting disagreements. All research members participated in developing the criteria and drafting the protocol for this systematic review. LF, LD and LW established the search strategy. DL, HL, CH and SM will independently accomplish the study selection, data extration and assess the risk of bias. HC,YH, LL and WL will perform the data syntheses. The subsequent and final versions of the protocol are critically reviewed, modified and authorized by all authors.

**Conceptualization:** Dongqiang Luo.

**Data curation:** Dongqiang Luo.

**Formal analysis:** Dongqiang Luo, Bin Liu.

**Funding acquisition:** Feng Liu.

**Methodology:** Bin Liu, Pengxin Wang.

**Project administration:** Dongqiang Luo, Lu Liu, Wanning Lan, Feng Liu.

**Software:** Dongqiang Luo, Bin Liu, Pengxin Wang.

**Supervision:** Pengxin Wang.

**Validation:** Pengxin Wang.

**Visualization:** Hui Liao.

**Writing – original draft:** Hui Liao, Shuxian Mao, Huicong Chen, Yuxin Huang, Lu Liu, Wanning Lan.

**Writing – review & editing:** Hui Liao, Shuxian Mao, Huicong Chen, Yuxin Huang, Lu Liu, Wanning Lan.

## References

[1] Seyed Hosseini E Riahi Kashani N Nikzad H. The novel coronavirus Disease-2019 (COVID-19): mechanism of action, detection and recent therapeutic strategies. Virology. 2020;551:1–9. 33010669 10.1016/j.virol.2020.08.011 PMC7513802

[2] Garg M Maralakunte M Garg S. The conundrum of “Long-COVID-19”: a narrative review. Int J Gen Med. 2021;14:2491–506. 34163217 10.2147/IJGM.S316708 PMC8214209

[3] Banerjee I Robinson J Leclézio A. Post COVID syndrome: a novel challenge and threat to international health. Nepal J Epidemiol. 2022;12:1215–9. 35974973 10.3126/nje.v12i2.46149 PMC9374107

[4] Fischer A Zhang L Elbéji A. Long COVID symptomatology after 12 months and its impact on quality of life according to initial coronavirus disease 2019 disease severity. Open Forum Infect Dis. 2022;9:ofac397. 35983269 10.1093/ofid/ofac397 PMC9379809

[5] Jinli Z Shuo Z Yunpu Q. COVID-19 sequelae and rehabilitation of traditional Chinese and western medicine. J Tradit Chin Med. 2021;62:2198–203.

[6] Williams JE Moramarco J. The role of acupuncture for long COVID: mechanisms and Models. Med Acupunct. 2022;34:159–66. 35832109 10.1089/acu.2021.0090 PMC9248327

[7] Trager RJ Brewka EC Kaiser CM. Acupuncture in multidisciplinary treatment for post-COVID-19 syndrome. Med Acupunct. 2022;34:177–83. 35821795 10.1089/acu.2021.0086 PMC9248328

[8] Chu L Huang F Zhang M. Current status of traditional Chinese medicine for the treatment of COVID-19 in China. Chin Med. 2021;16:63. 34315521 10.1186/s13020-021-00461-y PMC8314260

[9] Shamseer L Moher D Clarke M. Preferred reporting items for systematic review and meta-analysis protocols (PRISMA-P) 2015: elaboration and explanation. Bmj. 2015;350:g7647. 25555855 10.1136/bmj.g7647

[10] Page MJ McKenzie JE Bossuyt PM. The PRISMA 2020 statement: an updated guideline for reporting systematic reviews. Bmj. 2021;372:n71. 33782057 10.1136/bmj.n71 PMC8005924

[11] Moher D Liberati A Tetzlaff J. Preferred reporting items for systematic reviews and meta-analyses: the PRISMA statement. PLoS Med. 2009;6:e1000097. 19621072 10.1371/journal.pmed.1000097 PMC2707599

[12] Akbarialiabad H Taghrir MH Abdollahi A. Long COVID, a comprehensive systematic scoping review. Infection. 2021;49:1163–86. 34319569 10.1007/s15010-021-01666-x PMC8317481

[13] Taribagil P Creer D Tahir H. “Long COVID” syndrome. BMJ Case Rep. 2021;14:e241485. 10.1136/bcr-2020-241485 PMC8057566 33875508

[14] Aiyegbusi OL Hughes SE Turner G. Symptoms, complications and management of long COVID: a review. J R Soc Med. 2021;114:428–42. 34265229 10.1177/01410768211032850 PMC8450986

